# Comparison of Response to Rituximab Therapy in Adults with Refractory Symptomatic Immune Thrombocytopenia According to the Presence of Accessory Spleen

**DOI:** 10.3390/hematolrep14030030

**Published:** 2022-07-05

**Authors:** Fehmi Hindilerden, Ipek Yonal-Hindilerden, Mustafa Nuri Yenerel, Meliha Nalcaci, Reyhan Diz-Kucukkaya

**Affiliations:** 1 Division of Hematology, Department of Internal Medicine, Hamidiye School of Medicine, University of Health Sciences, Istanbul 34730, Turkey; 2 Division of Hematology, Department of Internal Medicine, Istanbul University Istanbul Medical Faculty, Istanbul 34130, Turkey; ipekyonal@hotmail.com (I.Y.-H.); mnyenerel@gmail.com (M.N.Y.); mnalcaci@hotmail.com (M.N.); 3 Department of Molecular Biology and Genetics, Istanbul University Faculty of Science, Istanbul 34170, Turkey; rkucukkaya@hotmail.com

**Keywords:** immune thrombocytopenia, rituximab, accessory spleen

## Abstract

No data exist for the association between the presence of accessory spleen after splenectomy and response to rituximab in immune thrombocytopenia (ITP). We investigated the relationship between accessory spleen presence and rituximab response in splenectomized ITP patients. Fifteen chronic refractory ITP patients were included. Four weekly doses of rituximab 375 mg/m^2^ were administered. All patients had undergone splenectomy before rituximab administration. Accessory spleen was detected in 5 of 15 patients (33.3%). Median age at diagnosis was significantly higher in patients with accessory spleen than those without accessory spleen (40 (range 25–68 years) and 26 (range 7–40 years), respectively; *p* = 0.049). There was a trend for older age at time of rituximab initiation in patients with accessory spleen compared to the other group (median 51 (range 43–75 years) and 42.5 (range 30–60 years), respectively; *p* = 0.066). Median follow-up duration was 96 months (range 40–98). We demonstrated a significant correlation between accessory spleen presence and older age. Accessory spleen presence correlated with higher platelet and WBC counts. We showed good inverse correlation between presence of accessory spleen and time to early response (ER) to rituximab while the rate of early response (ER), late response (LR), sustained response (SR) and overall response (OR) did not differ with respect to the presence of acessory spleen.

## 1. Introduction

Immune thrombocytopenic purpura (ITP) is an autoimmune disorder characterized by the premature destruction of platelets coated with IgG autoantibody by the reticuloendothelial system 1. Rituximab is a chimeric monoclonal antibody highly specific for the CD20 antigen that has the ability to deplete antibody-producing B lymphocytes. Rituximab has been administered in various autoimmune disorders including ITP [1]. The association between response rate to rituximab and splenectomy had been previously investigated [1,2,3,4,5,6,7]. In some studies, splenectomy status was found not to be a significant predictor of response to rituximab or the characteristics of the response including duration of response, type of response and time to achieve response [1,2,3,4]. In one previous study, splenectomized ITP patients tended to relapse earlier after rituximab therapy than nonsplenectomized patients [5]. Several studies reported that nonsplenectomized patients showed a higher early response (ER) rate compared to splenectomized patients [6,7]. To our knowledge, there are no previous data available on the association between the presence of accessory spleen after splenectomy and response to rituximab treatment. The overall initial response and sustained response (SR) rates to rituximab in 15 chronic refractory symptomatic ITP patients with a mean follow-up duration of 89 months had been previously published [8]. In the same group of splenectomized ITP patients, we herein compared the clinical and laboratory characteristics as well as response rates to rituximab according to the presence of accessory spleen.

## 2. Materials and Methods

### Patients and Study Design

We prospectively assessed the long-term follow-up data of median 96 months (range 40–98) of 15 patients diagnosed with chronic refractory ITP, all of whom had been treated with corticosteroids and splenectomy and various immunosuppressive agents including azathioprine, vincristine, danazol and mycophenolate mofetil. Exclusion criteria included the presence of severe infections, heart failure, pregnancy or chronic active hepatitis B infection. Rituximab 375 mg/m^2^ was administered IV once weekly for 4 weeks between November 2007 and March 2008. Rituximab was offered to these patients as an off-label treatment following the approval of the Ministry of Health. Ethics approval was obtained from the local Institutional Review Committee and a signed informed consent was obtained from all participants. Spleen scintigraphy was performed for the investigation of accessory spleen. CR was defined as any platelet count of at least 100.000/mm^3^, partial response (PR) as any platelet count between 30 and 100.000/mm^3^ and no response (NR) as any platelet count less than 30.000/mm^3^, or the presence of bleeding [9]. Early response (ER) was defined as a response within 42 days of rituximab infusion and late response (LR) as response occurring 42 days after initiation of rituximab. Overall response (OR) was the summation of ER and LR. SR was defined as response lasting for a minimum of 6 months [10,11]. Loss of response was defined as losing response to rituximab with any platelet count lower than 30.000/mm^3^, or the presence of bleeding and need of other therapy during follow-up. Time to response was defined as time from start of treatment until either CR or PR. Duration of response was defined as time from CR or PR until loss of CR or PR.

## 3. Statistical Analysis

Statistical analysis was performed using the SPSS version 21 (University of Sussex, Sussex, United Kingdom). Characteristics of patients were described as mean ± SD or median (range). Comparisons between groups were performed by chi-square test and Fisher’s exact test. The analysis of continuous variables among the groups was performed using the Mann–Whitney U test. Probability values of *p* < 0.05 were considered significant. The analysis of correlation between the presence of accessory spleen prior to rituximab and clinical and laboratory variables was performed according to Spearman’s rank correlation test.

## 4. Results

We prospectively analyzed 15 patients with chronic refractory ITP (13 females and 2 males), who were treated with rituximab at the Division of Hematology of the Istanbul University Istanbul Medical Faculty. All patients had a history of splenectomy prior to the administration of rituximab. In 5 out of 15 patients (33.3%), accessory spleen was detected before the initiation of rituximab.

Comparison of ITP patients according to the presence of accessory spleen prior to rituximab treatment.

Clinical and laboratory features of ITP patients according to the presence of accessory spleen were outlined in Table 1. Median age at diagnosis was significantly higher in patients with accessory spleen than those without accessory spleen (40 (range 25–68 years) and 26 (range 7–40 years), respectively; *p* = 0.049). Patients with accessory spleen showed a trend towards older age at initiation of rituximab compared to the other group (median 51 (range 43–75 years) and 42.5 (range 30–60 years), respectively; *p* = 0.066). The two groups showed no significant differences with respect to gender, actual age, age at time of splenectomy, hemoglobin level, WBC and platelet counts at diagnosis, initial response rate to corticosteroid therapy, number of previous therapies, interval between initial diagnosis and splenectomy, time from splenectomy to rituximab therapy and from diagnosis to initiation of rituximab, the rate of ER, LR, SR, loss of response, rate of OR to rituximab at 96th month, duration of ER and OR, time to response to rituximab of early and late responders and follow-up duration after rituximab therapy (*p* > 0.05). The frequency of comorbid diseases was higher in the presence of accessory spleen compared to the absence of accessory spleen, yet the difference showed no statistical significance (60% and 30%, respectively, *p* = 0.368). The presence of accessory spleen prior to rituximab showed moderate positive correlation with actual age, age at diagnosis, age at the time of rituximab therapy and at the time of splenectomy (r = 0.443, r = 0.525, r = 0.492 and r = 0.426, respectively). There was a good inverse correlation between the presence of accessory spleen and the time to early response to rituximab (r = −0.645). There was mild inverse correlation between the presence of accessory spleen and the rate of loss of response to rituximab (r = −0.32). There was mild inverse correlation between the presence of accessory spleen and interval between initial diagnosis and the start of rituximab as well as time from initial diagnosis to splenectomy (r = −0.361 and −0.347, respectively).

## 5. Discussion

Rituximab is still commonly used off-label as a second or third-line option in many countries. In the evidence-based guideline by Neunert C. et al., rituximab is recommended as second or third-line therapy for relapsed or refractory disease after corticosteroids, IVIG, or splenectomy (grade 2C evidence) [12]. In another expert consensus guideline, rituximab is presented as a valid option for chronic ITP [13]. Yet, a clear sequential order of splenectomy, rituximab, and TPO-receptor agonists after failure to achieve long-lasting remission on corticosteroids is not established [12,13]. Few studies have investigated the association between response rate to rituximab and splenectomy in ITP patients [1,2,3,4,5,6,7]. In this study, which included 15 chronic refractory splenectomized ITP patients, we aimed to compare the response rates of rituximab according to the presence of accessory spleen. Patients with chronic refractory ITP who initially responded to splenectomy but subsequently relapsed should be evaluated for the presence of accessory spleen, identified in 10% of adult patients with chronic refractory ITP in one series [14]. In 5 of our 15 patients (33.3%), accessory spleen was detected prior to rituximab administration. The comparison of the characteristics of our ITP patients according to the presence of accessory spleen showed a significant correlation between the presence of accessory spleen and older age.

In line with our observation, Marchese S. et al. reported that younger age poses risk for failure to radiologically identify accessory spleen and that the rate of detection of accessory spleen increases with older age [15]. Marchese S. et al. suggested that the recognition of small residual splenic tissue is far more challenging to detect in patients with a younger age, whose entire abdomen size is relatively smaller [15]. However, the size of our study population is limited, and this correlation may be a coincidence and our finding needs to be confirmed in larger scale studies.

The rate of ER was higher in our patients with accessory spleen compared to those with no accessory spleen, but this difference was not statistically significant (40% and 30%, respectively). The rate of LR was lower in the accessory spleen group; this difference was not significant (0 and 20%, respectively). In addition, no significant differences in terms of SR rate were observed between patients with and without accessory spleen (20% and 30%, respectively). A total of 80% of patients without accessory spleen and 50% of patients with accessory spleen lost their response to rituximab; there was a mild inverse correlation between the presence of accessory spleen and the rate of loss of response to rituximab. To our knowledge, this is the first study to report the relationship between the presence of accessory spleen and response to rituximab in refractory İTP. Yet, our study population size is limited to make strong recommendations. Several studies investigated the association between response rate to rituximab and splenectomy [1,2,3,4,5,6,7]. In the study by Braendstrup P. et al. including 35 patients, splenectomy was not a significant predictor of response to rituximab [2]. In the study by Cooper N. et al., splenectomy neither influenced the likelihood of response to rituximab nor the characteristics of the response including time to achieve response, duration of response or type of response [1]. In line with the aforementioned reports, several others found no correlation between response to rituximab and splenectomy status [3,4]. A small study by Schweizer C. et al. found a better response rate to rituximab in splenectomized patients [16]. Patel VL. et al. reported that there was no difference in SR rate for rituximab in adults with respect to splenectomy status [5]. However, in that study, splenectomized patients tended to relapse earlier than nonsplenectomized patients [5]. Two previous studies reported that nonsplenectomized patients showed a higher early ER rate compared to splenectomized counterparts [6,7]. It was hypothesized that intact spleen might be necessary to obtain ER to rituximab [6,7]. Pasa S. et al. suggested that splenectomy does not influence the response to rituximab, but it may lead to a longer duration to achieve response [17].

## 6. Conclusions

In our study, there was a good inverse correlation between the presence of accessory spleen and the time to ER to rituximab. In addition, there was a mild inverse correlation between the presence of accessory spleen and the rate of loss of response to rituximab. The current study is unique as it is the first study to provide clinical and laboratory characteristics as well as response rates to rituximab according to the presence of accessory spleen. Despite its limitations, our study provides novel, long-term, prospective, real-life follow-up data. In adults with refractory symptomatic ITP, multicenter studies with larger number of patients are required to confirm our findings and to determine the response to rituximab according to the presence of accessory spleen.

## Figures and Tables

**Table 1:** Characteristic features of 15 patients with chronic refractory ITP according to the presence of accessory spleen prior to rituximab treatment.

Chronic Refractory ITP Patients	Accessory Spleen Absent before Rituximab, n = 10	Accessory Spleen Present before Rituximab, n = 5	*p*
Age, at diagnosis, median y (range, min. to max.)	26 (7 to 40)	40 (25 to 68)	0.049
Actual age, median y (range, min.to max.)	51 (37 to 68)	58 (51 to 83)	0.098
Age, at the time of splenectomy, median y (range, min. to max.)	26.5 (10 to 45)	41 (25 to 71)	0.111
Age, at the rituximab infusion, median y (range, min. to max.)	42.5 (30 to 60)	51 (43 to 75)	0.066
Female, %	9 (90%)	4 (80%)	1
Hemoglobin at diagnosis, mean ± SD, g/dL	11 ± 0.66	11.6 ± 1.51	0.441
WBC count at diagnosis, mean ± SD, /mm^3^	8075 ± 2271	9390 ± 1755	0.178
Platelet count at diagnosis, mean ± SD, /mm^3^	9050 ± 4179	12,200 ± 3962	0.156
Response to initial corticosteroid, n (%)	10 (100%)	5 (100%)	0.608
R	6 (60%)	2 (40%)	-
NR	4 (40%)	3 (60%)	-
Accompanying diseases, n (%) Hypertension Diabetes mellitus	3 (30%) 1 (10%) 2 (20%)	3 (60%) 2 (40%) 1 (20%)	0.368
Number of previous therapies	3.6 ± 1.17	3.8 ± 0.83	0.75
Time from diagnosis to splenectomy, median m (range, min. to max.)	24 (7 to 60)	12 (2 to 36)	0.194
Time from diagnosis to rituximab therapy, median m (range, min. to max.)	228 (108 to 456)	132 (60 to 276)	0.177
Time from splenectomy to rituximab therapy, median m (range, min. to max.)	219.5 (62 to 401)	112 (43 to 257)	0.27
ER, n (%)	3 (30%)	2 (40%)	1
LR, n (%)	2 (20%)	0	0.524
Loss of response, n (%)	4/5 (80%)	1/2 (50%)	1
SR, n (%)	3 (30%)	1 (20%)	1
OR to rituximab at 96th month, n (%)	9 (100%)	4 (100%)	1
R (n, %)	1 (11.1%)	1 (25%)	-
NR (n, %)	8 (88.9)	3 (75%)	-
Duration of ER, median m (range, min. to max.)	52 (2 to 298)	51.5 (5 to 98)	0.767
Duration of OR, median m (range, min. to max.)	9 (2 to 98)	51.5 (5 to 98)	0.558
Time to response to rituximab of ER, median w (range, min. to max.)	2 (1 to 4)	1 (1 to 1)	0.197
Time to response to rituximab of LR, median w (range, min. to max.)	10 (8 to 12)	10 (8 to 12)	1
Follow-up period after rituximab treatment, median m (range, min. to max.)	97 (40 to 98)	96 (44 to 98)	0.688
Death, n (%)	1 (10%)	1 (20%)	1

NR; no response, R; response, ER; early response, LR; late response, SR; sustained response, OR; overall response, y; years, m; months; w; weeks.

## Data Availability

The data presented in this study are openly available in Harvard Dataverse at https://doi.org/10.7910/DVN/6LZJ2D (accessed date 28 June 2022).
